# Different learning aberrations relate to delusion-like beliefs with different contents

**DOI:** 10.1093/brain/awae122

**Published:** 2024-04-18

**Authors:** Rosa Rossi-Goldthorpe, Steven M Silverstein, James M Gold, Jason Schiffman, James A Waltz, Trevor F Williams, Albert R Powers, Scott W Woods, Richard E Zinbarg, Vijay A Mittal, Lauren M Ellman, Gregory P Strauss, Elaine F Walker, Jason A Levin, Santiago Castiello, Joshua Kenney, Philip R Corlett

**Affiliations:** Interdepartmental Neuroscience Program, Wu Tsai Institute, Yale University, New Haven, CT 06511, USA; Department of Psychiatry, Yale University, New Haven, CT 06511, USA; Department of Psychiatry, University of Rochester Medical Center, Rochester, NY 14623, USA; Department of Neuroscience, University of Rochester Medical Center, Rochester, NY 14623, USA; Department of Opthalmology, University of Rochester Medical Center, Rochester, NY 14623, USA; Department of Psychiatry, University of Maryland School of Medicine, Baltimore, MD 21228, USA; Department of Psychological Sciences, University of California Irvine, Irvine, CA 92617, USA; Department of Psychiatry, University of Maryland School of Medicine, Baltimore, MD 21228, USA; Department of Psychology, Northwestern University, Evanston, IL 60208-2710, USA; Department of Psychiatry, Yale University, New Haven, CT 06511, USA; Department of Psychiatry, Yale University, New Haven, CT 06511, USA; Department of Psychology, Northwestern University, Evanston, IL 60208-2710, USA; Department of Psychology, Northwestern University, Evanston, IL 60208-2710, USA; Department of Psychology and Neuroscience, Temple University, Philadelphia, PA 19122, USA; Department of Psychology, University of Georgia, Athens, GA 30602, USA; Department of Psychology, Emory University, Atlanta, GA 30322, USA; Department of Psychology, University of Georgia, Athens, GA 30602, USA; Department of Psychiatry, Yale University, New Haven, CT 06511, USA; Wu Tsai Institute, Yale University, New Haven, CT 06511, USA; Department of Psychiatry, Yale University, New Haven, CT 06511, USA; Wu Tsai Institute, Yale University, New Haven, CT 06511, USA; Department of Psychiatry, Yale University, New Haven, CT 06511, USA; Wu Tsai Institute, Yale University, New Haven, CT 06511, USA; Department of Psychology, Yale University, New Haven, CT 06511, USA

**Keywords:** delusions, prediction error, volatility, predictive coding, belief-updating, psychosis

## Abstract

The prediction error account of delusions has had success. However, its explanation of delusions with different contents has been lacking. Persecutory delusions and paranoia are the common unfounded beliefs that others have harmful intentions towards us. Other delusions include believing that one's thoughts or actions are under external control or that events in the world have specific personal meaning.

We compare learning in two different cognitive tasks, probabilistic reversal learning and Kamin blocking, that have relationships to paranoid and non-paranoid delusion-like beliefs, respectively.

We find that clinical high-risk status alone does not result in different behavioural results in the probabilistic reversal learning task but that an individual's level of paranoia is associated with excessive switching behaviour. During the Kamin blocking task, paranoid individuals learned inappropriately about the blocked cue. However, they also had decreased learning about the control cue, suggesting more general learning impairments. Non-paranoid delusion-like belief conviction (but not paranoia) was associated with aberrant learning about the blocked cue but intact learning about the control cue, suggesting specific impairments in learning related to cue combination. We fit task-specific computational models separately to behavioural data to explore how latent parameters vary within individuals between tasks and how they can explain symptom-specific effects. We find that paranoia is associated with low learning rates in the probabilistic reversal learning task and the blocking task. Non-paranoid delusion-like belief conviction is instead related to parameters controlling the degree and direction of similarity between cue updating during simultaneous cue presentation.

These results suggest that paranoia and other delusion-like beliefs involve dissociable deficits in learning and belief updating, which, given the transdiagnostic status of paranoia, might have differential utility in predicting psychosis.

## Teaser

Rossi-Goldthorpe *et al.* study the behavioural and computational mechanisms of delusion-like beliefsthe strongly held erroneous beliefs that characterize psychotic illnesses—and find that paranoid and non-paranoid delusion-like beliefs involve dissociable deficits in learning and belief updating.

## Introduction

Delusions—the fixed false beliefs that characterize psychotic illnesses such as schizophrenia (but also bipolar disorder, depression and neurological and autoimmune illnesses)—represent profound departures from consensual reality. They have yet to yield entirely to empirical investigation, and, although they appear to be readily treated with antipsychotic drugs that block dopamine D_2_ receptors, many patients (≤50%) do not experience symptom resolution. Furthermore, we lack a coherent account that connects the phenomenology of delusions (what it is like to experience them) with the mental and neural processes that underwrite them and the social factors that form and foment them. That is the promise of computational psychiatry.^1^ It has begun to deliver, but there is substantial distance yet to cover. Here, we try to address the puzzle of the contents of delusions with computational tools.

One influential theory of delusions argues that they result from aberrant prediction error signals. Prediction errors are the mismatches between expectation and experience that drive learning. They are encoded in dopamine signals in the midbrain and striatum and in the brain more broadly.^2^ When they happen aberrantly, delusions can result.^3-5^ Such signals can direct learning to irrelevant events^6^ but they also may encourage overarching and spurious beliefs in volatility.^7,8^ However, it remains unclear whether these two aspects of prediction error processing are coincident responses to the same errors and, furthermore, whether these responses relate to delusions with different contents. These are the issues we attempt to address here.

In this paper, we move the prediction error theory further in three ways: (i) we examine delusion-like beliefs with different contents that fall on the continuum from health to illness, paranoia or persecutory delusion; (ii) we do so in healthy people and in people at clinical high risk for psychosis (CHR-P) and matched clinical controls; and (iii) we use two behavioural tasks (with attendant computational models) in an effort to refine the relationships between learning mechanisms and psychotic symptoms and progress the prediction error theory of delusions, with a particular focus on delusion-like belief contents.

One key phenomenon that emphasizes the role of prediction error in learning is the Kamin blocking effect.^9^ When a novel cue (e.g. a tone) is paired with a stimulus (e.g. a light) that already predicts an outcome (electric shock), the pretrained cue (the light) ‘blocks’ new learning about the novel cue (the tone). This is not the case in people prone to delusion-like beliefs, who evince brain prediction error signals during the blocking trials and learn about the blocked cue.^6^ Likewise, in rodents, optogenetic^10^ and chemogenetic^11^ techniques can be used to induce prediction error signals at the time of blocking trials in the midbrain and dorsomedial prefrontal cortex, respectively, engendering learning about the blocked cue. Such learning also occurs with behavioural pharmacological model psychoses in rats.^12^ Furthermore, brain prediction error signals are engaged inappropriately in people with delusions and are correlated with the severity of delusions.^3^ Similar relationships have been observed in many task contexts and in model psychoses wherein people are administered a drug that engenders a psychotic state transiently and reversibly. In these settings, again aberrant prediction errors have been observed, and again the magnitude of these aberrant signals correlates with the severity of delusion-like beliefs that participants experience.^13,14^ Delusions might form under the influence of aberrant prediction error signals, registered inappropriately, which drive attention and learning towards irrelevant stimuli.^4^ These effects were present regardless of the content of delusions.

Aberrant prediction error signals might also yield an overarching belief that the world is unpredictable, unstable and sinister.^7^ This would manifest as an altered sensitivity to volatility (the rate of change of the state of the task or the world) during learning in patients with psychosis. Sense of volatility has been studied using probabilistic reversal learning tasks. Given a choice between three options for points reward (or loss), people with schizophrenia tend to switch choices even after a win.^15-17^ This suboptimal behaviour may be particularly prevalent in people who are paranoid, rather than connoting delusions more broadly.^18^ Furthermore, paranoia mediates the relationship between worry and task-derived volatility beliefs in patients with schizophrenia.^19^ This is notable because worry is a key target for cognitive behavioural therapies for persecutory delusion.^20^ Understanding the underlying mechanisms of paranoia and other delusion-like beliefs will inform the future application and development of this approach.

The present work represents a key next step in the development of prediction error theory of delusions (and delusion-like beliefs). Can one explain delusion contents without appeal to content-specific dysfunction? Our prior work suggests that domain-general prediction errors are correlated with delusions with varied contents^3,6,21^ and that precision weighting of general prediction errors relates to paranoia.^15,16,19^

Here, we examine both aspects of prediction error, in the same people, for the first time. We sought to examine whether these aspects of prediction error processing (unselective learning and aberrant precision weighting, cast as aberrant learning rates or priors on volatility) were associated with delusion-like beliefs with different contents. We administered two cognitive tasks with differing demands on prediction error processing to the same participants for the first time: a Kamin blocking task, which requires selective learning about competing cues with deterministic consequences^6^; and a probabilistic reversal learning task, which requires belief updating under uncertainty in a volatile task environment.^16^ We examined the associations between behavioural performance in the tasks, paranoia and other delusion-like beliefs. Furthermore, we designed and fitted a computational model for the Kamin blocking task and fitted the hierarchical Gaussian filter (HGF) to the probabilistic reversal learning (PRL) task. Both models aimed to explain task performance, after which we examined the associations between computational model parameters and symptom experiences, to make new, differential, mechanistic claims about paranoia versus other delusions.

Prior work suggests that distress is the variable that distinguishes delusional patients from those with esoteric odd beliefs,^22,23^ and that weaker Kamin blocking is correlated with distress regarding delusion-like beliefs.^6,21^ However, clinical delusions are typically held with incorrigible conviction.^22,23^ To be convinced of these bizarre beliefs ought to be distressing,^24^ and across a series of studies, Schmack and colleagues^25-27^ have found that Peters *et al.* Delusions Inventory (PDI) conviction relates to a weakening of the effect of past regularities on current processing in people with delusions and delusion-like beliefs. Hence, we predicted that delusion-like belief conviction would be associated with weaker Kamin blocking.

In the present work, we computed the mean conviction associated with each belief endorsed on the PDI, excluding paranoid beliefs. We examined the blocking and reversal learning behaviour of people who were particularly convinced of their non-paranoid delusional beliefs on the PDI-21 and compared their patterns of behavioural responding (and computational model parameters) with those of people who endorsed high versus low paranoia on the Revised Green *et al*. Paranoid Thoughts Scale (R-GPTS). We note that is possible to have both high paranoia and high conviction in non-paranoid beliefs, but nevertheless, we sought the patterns of behaviour associated with paranoid and non-paranoid delusion-like beliefs.

## Materials and methods

Data collection for this multi-site study commenced in late 2020. Study sites included the following: Northwestern University; University of Maryland-Baltimore County; Yale University; University of Georgia; Temple University; Emory University; and the University of California Irvine. Owing to the coronavirus disease 2019 (COVID-19) pandemic, and related safety and social distancing policies, it was necessary to conduct the study remotely. All screening, baseline and follow-up sessions were conducted via Zoom or Webex [i.e. Health Insurance Portability and Accountability Act-compliant secure videochat platforms], and all behavioural tasks were implemented over the internet, via an in-house bespoke software platform. Although remote, each participant was guided through tasks by research assistants supervising the sessions.

This work was approved by Northwestern University as the Institutional Review Board of record and acknowledged by the Institutional Review Boards of all other participating sites. Written informed consent was provided by all participants. All work was conducted in accordance with the Declaration of Helsinki.

### Blocking task

In our food-allergy causal belief learning task, participants are asked to imagine that they are allergists and to learn the causes of allergic reactions in a fictitious patient. In each trial, they are shown a meal consisting of one or two different foods that the patient had eaten and are then given feedback regarding whether that meal caused an allergy. Their task is to learn to predict the outcome of each meal. The task is divided into three phases: the learning phase (10 repetitions of each stimulus); the blocking phase (six repetitions of each stimulus); and the test phase (six repetitions of each stimulus). The seven categories of stimuli are shown in Supplementary Table 1. Prior learning that one food (i.e. bananas) causes the allergy prevents (blocks) learning that another novel food (i.e. mushrooms) could also cause an allergy. In later trials, when participants receive feedback that mushrooms cause allergy, a prediction error brain response is observed.^6^ Each trial of the blocking task results in a response that ranges between −1 and 1, scaled by confidence.

Using the first trial of the testing phase, the blocking score was calculating as the response to the blocked cue (B2−), and the control score was calculated using the response to the blocking confirmation control cue (D1+).

### Probabilistic reversal learning task

This three-option PRL task,^15,16^ wherein participants learn and update reward associations in light of variable outcomes, owing to anticipated but uncertain changes in reward between options (reversal events, expected volatility) and unanticipated changes in the underlying probabilities themselves (contingency transition, unexpected volatility), challenges participants to form and update beliefs about the value of each option and the volatility of the task environment. Participants choose between three decks of cards with hidden reward probabilities, selecting a deck on each turn and receiving positive or negative feedback (+100 or −50 points, respectively). They are instructed to find the best deck, with the caveat that the best deck might change. Undisclosed to participants, reward probabilities switch among decks after selection of the highest probability option in 9 of 10 consecutive trials (‘reversal events’).^16,17,19,28^ There are 160 trials in total, with 80 trials in each block (reward contingencies are detailed in the Supplementary material).

We calculated the win-switch rate (WSR; switches after winning/total wins) for each participant using their choices from the PRL, allowing us to measure the rate of erratic switching.

**Figure 1:**
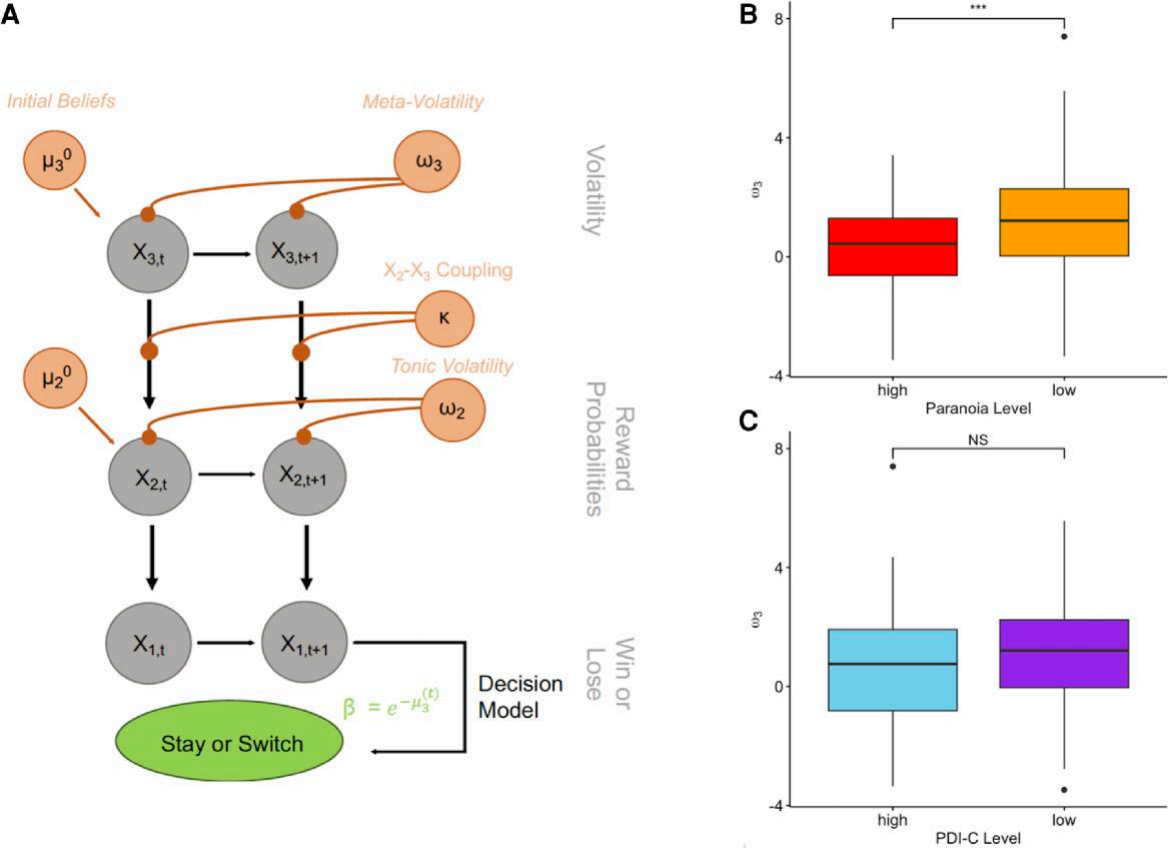
**Hierarchical Gaussian filter (HGF) model and parameters.** (**A**) Schematic diagram of HGF adapted from Suthaharan *et al*.^16^ Computed beliefs are in grey circles and fitted parameters in orange circles. Beliefs are updated every trial and produce the choice of cards. (**B**) The parameter $\omega_{3}$, which represents the rate of change for the volatility belief, is higher in individuals with high paranoia. (**C**) There was no difference in $\omega_{3}$ when comparing by non-paranoid delusion-like belief conviction level. ***P ≤ 0.001, NS: *P* > 0.05. PDI-C = Peters *et al*. Delusions Inventory-conviction.

As before,^15,16^ we fitted an HGF to PRL behaviour, comprising a perceptual model, which captures participants’ task beliefs, and a response model that governs how beliefs are converted into choices (Fig. 1A). The perceptual model has three hierarchical layers of belief about the task. The layers interact and influence one another through learning rate parameters. At the highest level (Level 3), the model captures beliefs about changes in the task environment (how are values of the choices changing over time?). Level 2 characterizes beliefs on reward probabilities (i.e. the tendency of a choice to be rewarding). Level 1 characterizes task reward feedback (i.e. win or loss). These three levels of belief are then integrated and fed through a sigmoid response function to produce a decision (whether to stay with the same card deck or switch to a different one).

### Blocking modelling

We adapted a reinforcement learning framework that separately tracks and updates values for different cues using a weight matrix.^29^ We chose to use an elemental representation for compound stimuli (one column in the weight matrix per cue). In a trial where multiple cues are present, weights for both present cues are updated. A single static learning rate parameter, *α*, is used for updating all the cues. A flexible integration rule using free parameter *γ* allows for compound cue prediction ranging from additive to maximum only.

Some individuals appear to engage in counterfactual learning: if they learn that the primary cue causes allergy and observe that the compound stimulus causes allergy, they then assume that the secondary cue does not cause the allergy (they have no information about the secondary cue and thus should not learn about it). We incorporate this into the model by adding an additional parameter, *λ*, that sets the learning asymptote for the secondary cue. This can range from one (driving learning about the secondary cue towards the observed outcome attributed to the primary cue) to minus one (counterfactual learning about the secondary cue). An additional feature noted in the data was that individuals appeared to ‘forget’ the values of the cues when the task shifted to a new phase. Of note, there was no explicit separation between the phases, but the cues did change, signifying a new phase. To incorporate this feature into the model, we introduce a ‘forgetting’ parameter that decays the weights on the first trial of a new phase, *η*. Detailed equations for weight updating and prediction are found in the Supplementary material.

We used maximum likelihood estimation to estimate model parameters for each individual using their choices. We assumed that the model prediction was used directly as the model response value and calculated the log-likelihood assuming a Gaussian distribution centred at $\hat{r}$, using a variance computed using the mean difference between model responses and the responses of a participants^30^ in order to reduce model complexity. Models were fitted using the mfit package,^29^ with a total of 25 initial parameter sets; the best-fitting parameter was chosen from these fits.

We used Bayesian model selection to deduce which model was more likely to have generated the data (protected exceedance probability^31^). Protected exceedance probabilities are given in Supplementary Table 2, along with the model variations tested. We tested versions that included removing the flexible integration rule, which removes *γ* (RW4 and RW5), removing the ‘forgetting’ parameter (RW3) and adding a decision noise parameter for the variance of the Gaussian in the log-likelihood (RW1). Ensuring that the entire model space can capture the key behavioural metrics is crucial for modelling.^32^ RW2 was found to be the winning model.

### Summary of modelling

In what follows, we will model choice behaviour (in the blocking and reversal learning tasks) using a reinforcement learning model and an HGF, respectively. Although these models have different functional forms, they both relate to our central thesis that delusions arise from aberrant predictive processing; specifically, a failure to instantiate the precision of prediction errors that drive belief updating, learning and subsequent choice behaviour.

In brief, precision refers to the reliability or confidence afforded the neuronal representation of prediction errors, such that precise prediction errors exert a greater influence on inference and learning. In the reinforcement learning models, we will consider three types of precision. First, we will consider the precision (*α*) of prediction errors that drive learning (in a Q-learning context). This formulation of precision highlights its role as a learning rate. Second, we will consider the precision (*γ*) of secondary cues, relative to primary cues. This reflects the key role of precision in (e.g. multisensory) integration of various sources of evidence. Furthermore, we incorporate *λ*, which gates the impact of blocking trials on beliefs about the blocking (primary) and blocked (secondary) cues, such that counterfactual inference can implement learning that the blocked cue does not cause the allergy (not merely that it causes allergy less). Finally, we will consider the precision (*η*) of latent dynamics in terms of a forgetting parameter. When dynamics have a low precision (i.e. high variance), they are volatile.

This brings us to the key role of the HGF that models precision in terms of (inverse) volatility, within a hierarchical generative model. This hierarchical model clarifies how (precision-weighted) prediction errors at various levels of the hierarchy are used to estimate precision in a biologically plausible fashion.

### Statistics

Statistical analyses were performed with an α of 0.05 and two-tailed *P*-values in Rstudio v.1.3.959.

Repeated-measures ANOVAs were used to analyse group differences for WSR, HGF parameters, and the difference between blocking and control scores. For *post hoc* testing, *t*-tests were used. Effect sizes ($\eta_{p}^{2}$ and Cohen's *d*) were computed using the *effectsize* package in R.

## Results

### Sample characteristics

Our final sample consisted of 452 individuals [clinical high risk (CHR) = 181, help-seeking control (HSC) = 161 and healthy control (HC) = 110; Supplementary Table 3]. Of those, 65 met the clinical cut-off for high paranoia using the R-GPTS, whereas the remaining 387 were considered low paranoia. Given that the PDI does not have established cut-offs, we split the participants into groups based on the relative PDI-conviction (PDI-C) values as follows: anyone above the 75% quantile for the non-paranoid PDI conviction scores was labelled as high PDI-C (*n* = 113), with anyone below that value labelled as low PDI-C (*n* = 339). These groupings are not distinct; there is overlap between the paranoia groups and the PDI groups. We note that the PDI is a tool used on the continuum from health to delusion. Strictly, the beliefs we focus on this report are delusion-like, particularly given that even our CHR participants are at risk of psychosis and not (yet) delusional. Nevertheless, studying delusion-like beliefs is relevant to delusions and common practice in the computational psychiatry of psychosis (e.g. see Schmack *et al.*^25^ and Teufel *et al.*^33^).

### Probabilistic reversal learning

**Figure 2:**
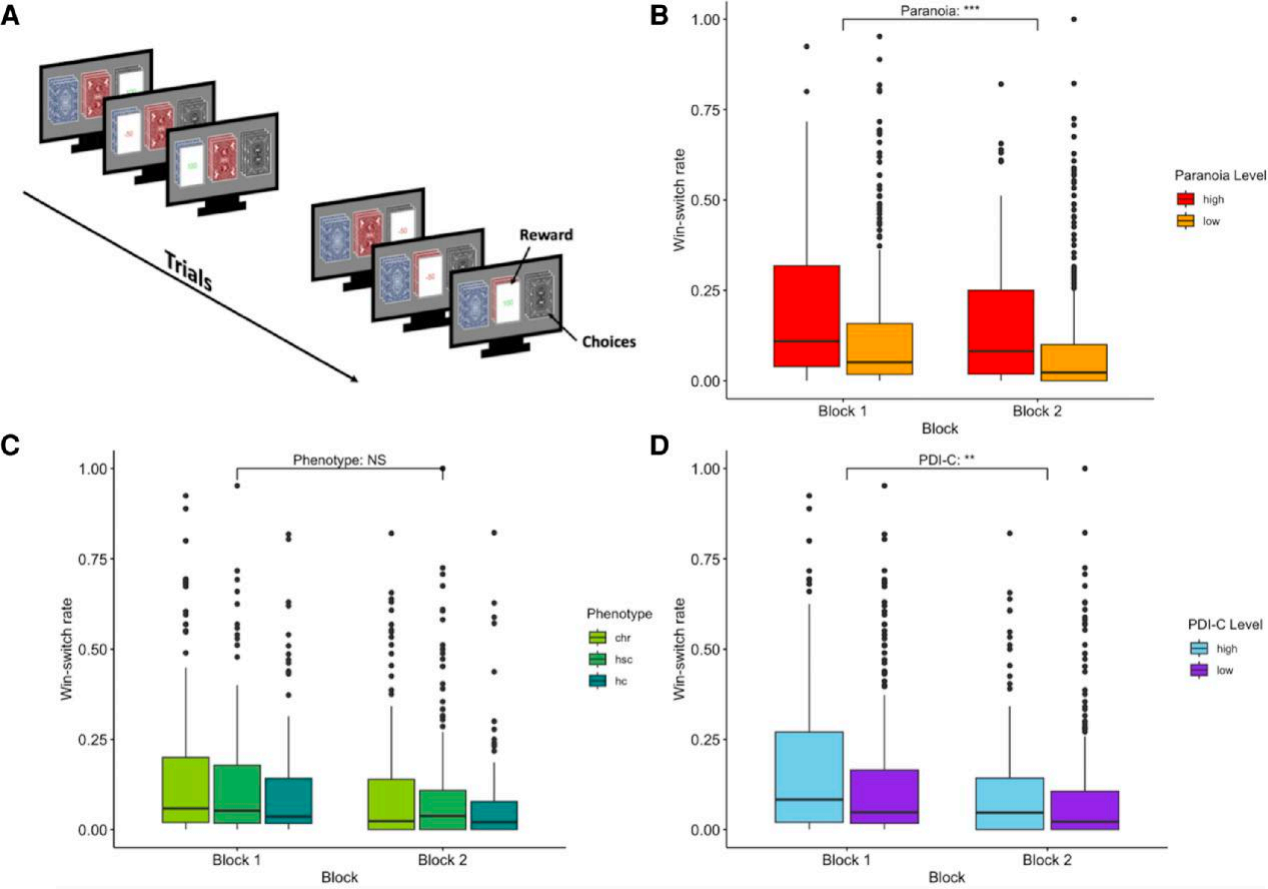
**Win-switch rates in the probabilistic reversal learning (PRL) task.** (**A**) Schematic diagram of the PRL task. Participants choose the best option between three decks of cards that leads to a probabilistic reward. (**B**) Win-switching was enhanced in participants with high paranoia. (**C**) Win-switching was not different when comparing clinical phenotype groups (CHR = clinical high risk; HC = healthy control; HSC = help-seeking control). (**D**) Win-switching was higher in individuals with high non-paranoid delusional conviction. PDI-C = Peters *et al*. Delusions Inventory-conviction. **P ≤ 0.01; ***P ≤ 0.001; NS: *P* > 0.05.

Paranoia was, again,^16,28^ associated with erratic win-switching on PRL, replicating prior work [*F*(1,450) = 16.05, *P* < 0.0001, $\eta_{p}^{2}$ = 0.03; Fig. 2A]. In contrast, several measures of depression and hallucinations were unrelated to WSR (Supplementary Table 4). Probabilistic reversal learning involves decision-making under uncertainty about which option to choose and whether the options have recently changed or even reversed in their value. By studying people's choices with computational modelling, we can infer the latent processes (or beliefs) that led to those choices. This rests on the premise that people form and update a number of beliefs about the properties of the task, which help to guide their choices.

HGF model analysis revealed that paranoia and win-switch behaviour manifest as poor learning from volatility, as before; specifically, as less flexible belief-updating in response to volatility [captured by the $\omega_{3}$ parameter, see the ‘Materials and methods’ section; *F*(1,448) = 17.71, *P* < 0.0001, $\eta_{p}^{2}$ = 0.04; Fig. 1B; no other model parameters were significantly different]. We note that previous model parameter recovery has been suboptimal. Here, we broadened the prior distributions on model parameters (Supplementary Table 5) and achieved much more satisfactory parameter recovery at Level 3 [Supplementary Table 6 and Supplementary Fig. 1; note that recovery of some parameters at Level 2 is poor; initial beliefs about rewards ($\mu_{0}^{2}$) and phasic learning rate (*κ*), given that these parameters also influence what happens at Level 3, we should be cautious in interpreting our Level 3 findings]. As before, paranoia is the purview of Level 3. Participants’ learning about task changes (meta-volatility learning rate) was significantly related to their paranoia. It was not significantly related to delusion-like beliefs more broadly [*F*(1,448) = 3.37, *P* = 0.07, $\eta_{p}^{2}$ < 0.01; again, no other parameters were significantly different].

### Kamin blocking

**Figure 3:**
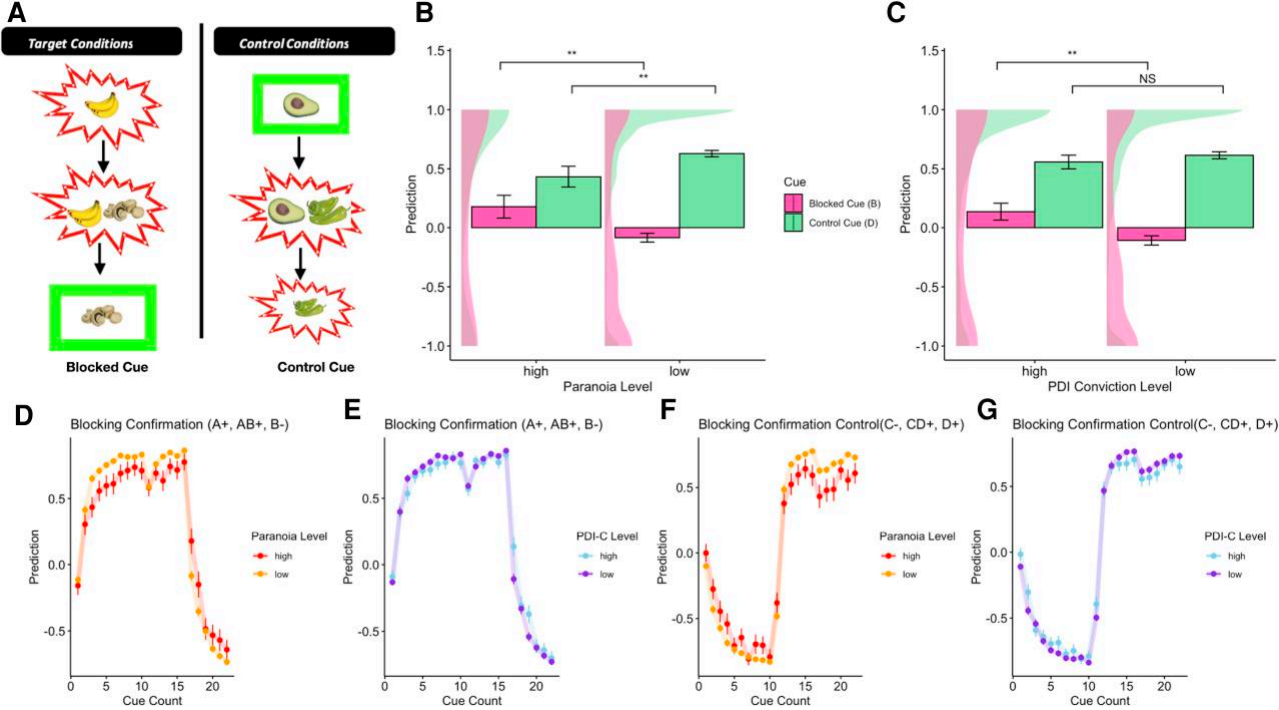
**Kamin blocking task and behavioural results.** (**A**) Schematic diagram of blocking and control trial types throughout the task. In Phase 1, the blocking stimulus (banana) causes the allergy (red outline). The blocking stimulus is then paired with a novel stimulus (mushrooms) in Phase 2, and the compound causes allergy. The blocking score is then determined using the response to the blocked cue (mushrooms) on the first trial of Phase 3. To produce the control score, a control cue (avocado) does not cause allergy (green outline) in Phase 1 and is then paired with a novel cue (peppers) in Phase 2; the compound causes allergy. The control score is the response to the novel control cue (peppers) in Trial 1 of Phase 3. (**B**) Individuals with high paranoia have lower control scores in comparison to participants with low paranoia and to impaired blocking (higher responses to the blocked cue). Error bars show the standard error of the mean (SEM). (**C**) Participants with high non-paranoid delusion-like belief conviction have impaired blocking (increased responding to the blocked cue) but have intact responding to the control cue. Error bars show the SEM. (**D**–**G**) Averaged responses for different trial types over the course of the task. Error bars show the SEM. Each trial type was shown 10 times in Phase 1 and six times in Phases 2 and 3. Phase 2 then occurs at the 11th time the trial type appears. Blocking trials were calculated using the first trial from Phase 3, which is the 17th time the trial type is shown. (**D**) Mean responses to the blocking confirmation trials (A_2_+, A_2_B_2_+ and B_2_−) over the course of the task for participants with high or low paranoia. (**E**) Mean responses to the blocking confirmation trials (A_2_+, A_2_B_2_+ and B_2_−) over the course of the task for participants with high or low non-paranoid delusional conviction. (**F**) Mean responses to the blocking confirmation control (C_1_−, C_1_D_1_+ and D_1_+) cues split by paranoia level. (**G**) Mean responses to the blocking confirmation control (C_1_−, C_1_D_1_+ and D_1_+) cues split by non-paranoid delusion-like belief conviction level. PDI-C = Peters *et al*. Delusions Inventory-conviction. **P ≤ 0.01; NS: *P* > 0.05.

Blocking was also impaired in people with high paranoia [Paranoia × Cue type: *F*(1,450) = 5.85, *P* < 0.001, $\eta_{p}^{2}$ = 0.03; Fig. 3B] and in people with high conviction in non-paranoid delusion-like beliefs [PDI-C × Cue type: *F*(1,450) = 3.81, *P* < 0.01, $\eta_{p}^{2}$ = 0.02; Fig. 3C]. This would suggest, by the traditionally used metric of the difference between the control cue and blocked cue, that both paranoia and non-paranoid delusion-like belief conviction are associated with impaired blocking, but the contributions of the two responses vary depending on belief content. Both were associated with increased responding of ‘allergy’ for the blocked cue [paranoia: *t*(450) = 2.67, *P* < 0.01, Cohen’s *d* = 0.36, 95% confidence interval (CI): (0.07, 0.45); PDI-C: *t*(450) = 3.1, *P* < 0.01, Cohen’s *d* = 0.33, 95% CI: (0.09, 0.4); Fig. 3B–E]. However, the responses to the control cue were different between the two cases: control cue learning was intact in people who had high conviction in their non-paranoid delusion-like beliefs [high PDI-C versus low PDI-C: *t*(450) = −0.92, *P* = 0.36, Cohen’s *d* = −0.1, 95% CI: (−0.18, 0.06); Fig. 3C and G]; in paranoid people, control learning was also impaired [high versus low paranoia: *t*(450) = −2.6, *P* = 0.01, Cohen’s *d* = −0.35, 95% CI: (−0.34, 0.05); Fig. 3B and F]. Looking only at the control-blocked cue difference does not allow for differentiating these two patterns. Neither control cue score nor blocked cue score was associated with depression or hallucinations (Supplementary Table 3).

#### Computational model of blocking

On surveying the literature, we were surprised by the absence of individual-level model fitting to human Kamin blocking behavioural data, particularly in tasks with binary outcomes.^34^ This is notable because the blocking phenomenon was the impetus to create the Rescorla–Wagner (RW) learning rule; given that blocking emphasizes that there is more to learning than contiguity between the cue and the outcome, the outcome must be surprising for learning to occur.^35^ We set out to estimate model parameters from participant behaviour and to relate those parameters to paranoia and other non-paranoid delusional beliefs.

**Figure 4:**
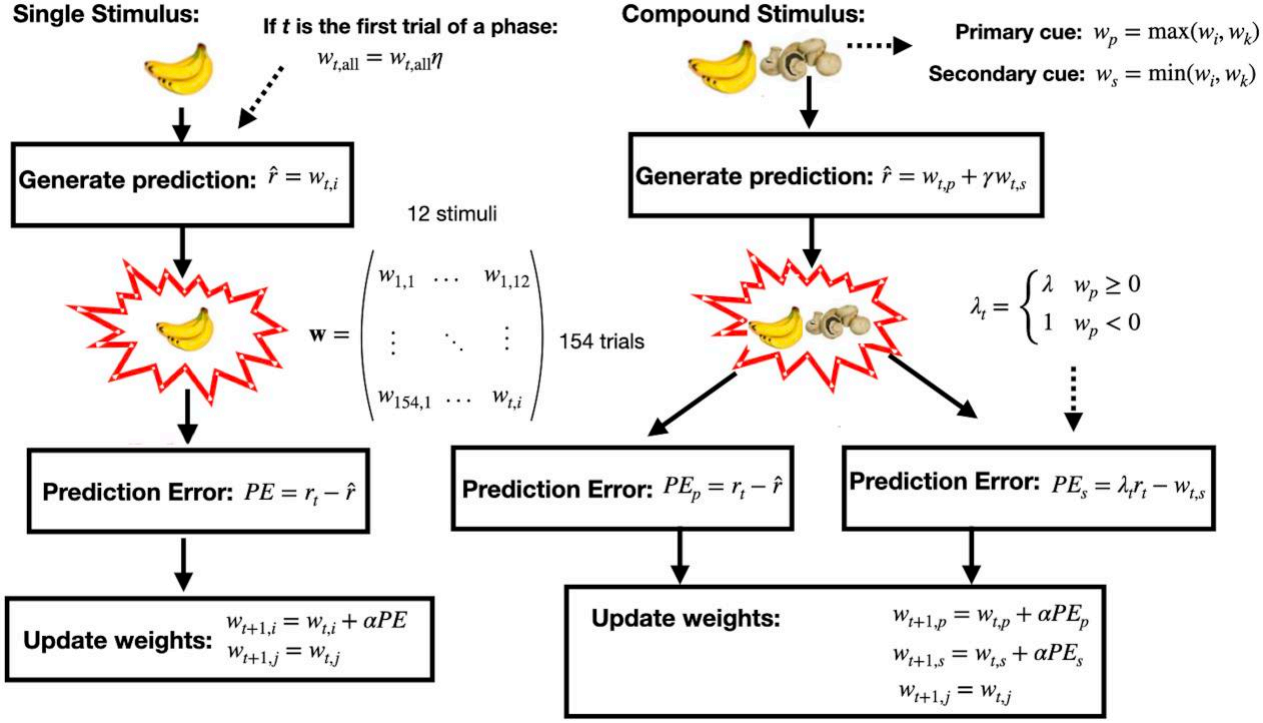
**Schematic diagram of the modelling framework to model responses to the blocking task.** Single and compound stimuli are updated differently. The prediction ($\hat{r}$) is used as the choice in this model.

Initially, we sought a computational model of the blocking phenomenon that could capture the spectrum of responses to the blocked cue (Fig. 4). Of course, prediction error is a key component for explaining Kamin blocking^35^ and delusions.^3,4^ Aberrant prediction errors can drive attention and learning towards the redundant stimulus.^6^ However, we sought to capture the full range of behavioural responses that people evince across the task phases to explore the various possibilities for how cues integrated and updated to produce the observed range of behaviour responses.^36^ Furthermore, we aimed for a more comprehensive account of aberrant prediction error; how exactly is the signal aberrant? What are the consequences of aberrant prediction error?

We began with a classical RW model, in which each cue has an associated weight that is used to generate the response in a trial.^29^ The weight matrix is updated using the classic RW learning rule (Fig. 4), resulting in only the weights corresponding to cues that are present in a given trial being updated. For trials with a single cue, the weight associated with that cue is simply the model-generated response. Trials in which multiple cues appear concurrently present a more complex situation; two cues are combined to produce the overall estimation for the compound stimulus. An assumption of the classical RW model is that associative strengths (in our model, the weights) of cues are additive^35^; however, in a task where additivity is not pretrained (outcomes are binary), attributing causal power to the cue that is more likely to cause the allergy (cue-1) and disregarding the other cue (cue-2) might be more appropriate.^37,38^ Rather than making an assumption regarding cue integration, we use a flexible cue integration rule that allows for a range of cue combination ranging from additivity (cue-1 + cue-2) to maximum cue[max(cue-1, cue-2)] only^38^ (Fig. 4).

**Figure 5:**
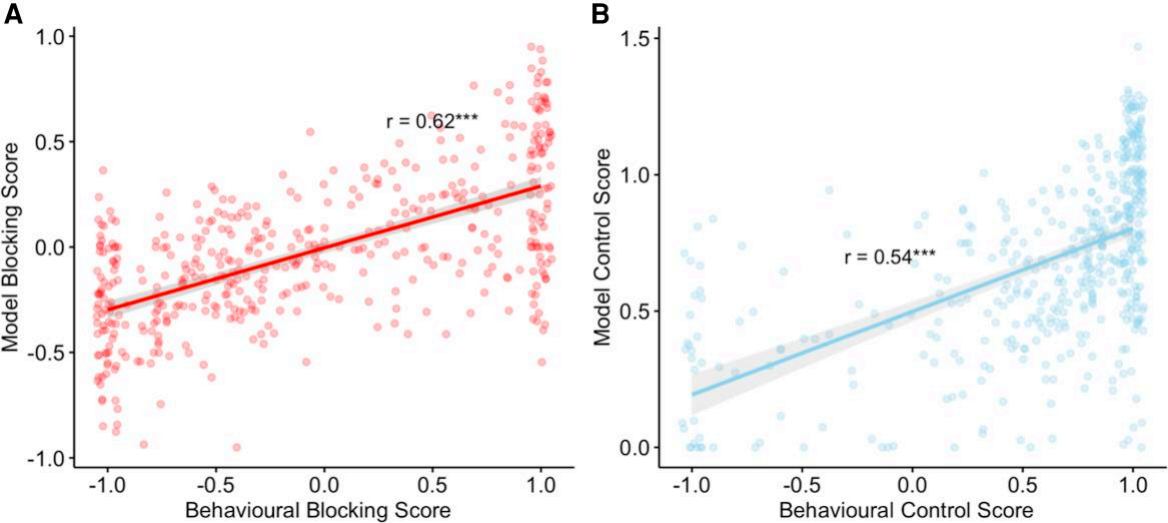
**Comparison of behavioural blocking and control scores and model-estimated scores using cue weights from the model.** (**A**) Behavioural blocking and model blocking scores were strongly correlated. (**B**) Control score and model control scores were also correlated. ***P ≤ 0.001.

Our choice of a flexible cue integration rule necessitates the use of non-selective prediction errors in the updating of the cues in a compound trial.^39^ If individuals are using only one cue (primary cue) in their responses, we avoid the assumption that both cue values (primary and secondary) are updated in the same way. To allow for the range of observed behaviour, we modify how the outcome is attributed to the secondary (non-causal) cue; individuals can attribute the outcome entirely to the primary cue, meaning that the secondary cue is not updated. In this way, we can still see the classical blocking effect (where learning about the novel cue is ‘blocked’ by the fact that the already-known cue causes the allergy) even if participants are not actually adding the values of the stimuli.^40^ Given that outcomes are not additive in this framework, we believe that this is more appropriate to model the blocking effect. Additionally, this framework provides the chance to describe the behaviour of individuals who neither display blocking nor associate the blocked cue with the known cue: we and others have observed that blocked cues (Fig. 3B and C) can be rated as preventative of the outcome (rather than merely less causal).^36^ This suggests that learning about one cue could engender counterfactual inference about another cue,^41,42^ as in retrospective revaluation^43^ or blocking attributable to propositional inference.^21^ The behaviour of interest here (blocking) was assessed with only a single trial in the test phase, whereas the model was fitted to the entirety of the task (154 trials). To assess how well the model captured the behaviour of interest, we computed model blocking scores (the response of the model to the blocked cue at the beginning of Phase 3) and compared the correlation with the behavioural blocking scores of the participants. The model performed well, particularly for estimating scores to the blocked cue in addition to the control cue [blocked cue: *r* = 0.62, *P* < 0.0001, 95% CI: (0.56, 0.67); control cue: *r* = 0.54, *P* < 0.0001, 95% CI: (0.47, 0.60); Fig. 5].

**Figure 6:**
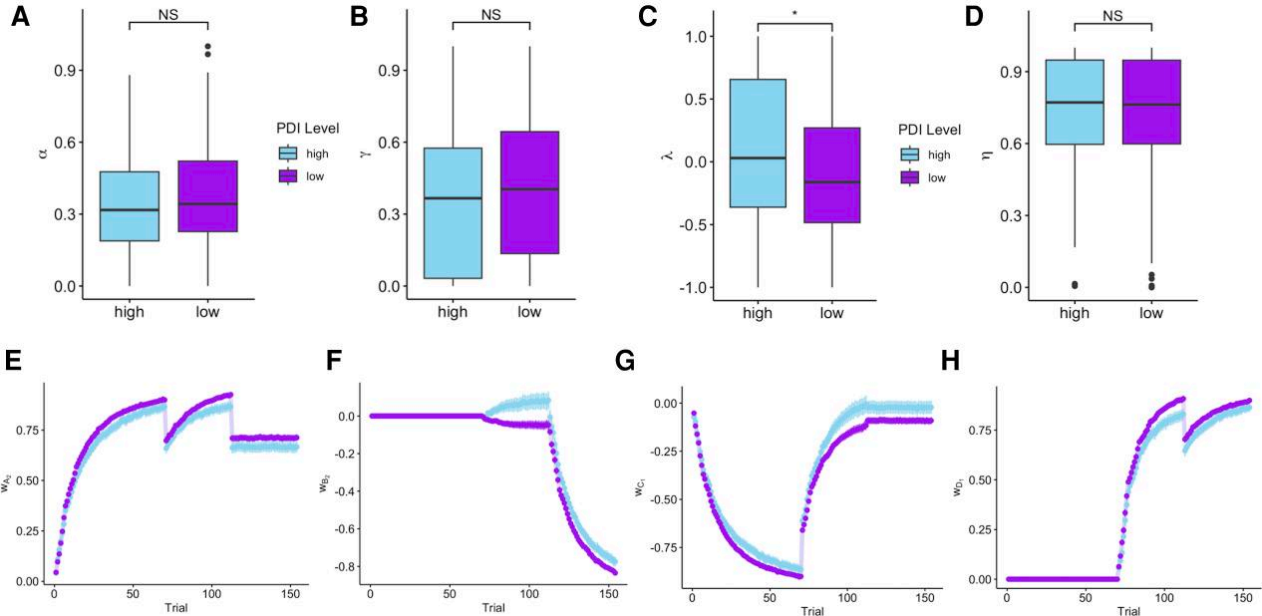
**Model parameters by non-paranoid delusion-like belief conviction levels.** (**A**) Learning rate (*α*) was not significantly different between groups. (**B**) The integration rule parameter (*γ*) was also not significantly different between the groups. (**C**) The learning asymptote parameter (*λ*) for the secondary cue was significantly higher in the high PDI-C group, reflecting less counterfactual learning and a higher degree of similarity in updating between the secondary and primary stimuli. (**D**) There was no significant difference in cue decay at the onset of a new phase. (**E**–**H**) Time series for averaged model cue weights. Error bars show the standard error of the mean. (**E**) Weights for the blocking cue were similar for the two groups. (**F**) There was greater separation during Phase 3 for the weight of the blocked cue; the weight increased during A_2_B_2_+ pairing for the high PDI-C group. (**G** and **H**) Weights for the control cues were similar when grouping by PDI-C level. *P ≤ 0.05; NS: *P* > 0.05.

**Figure 7:**
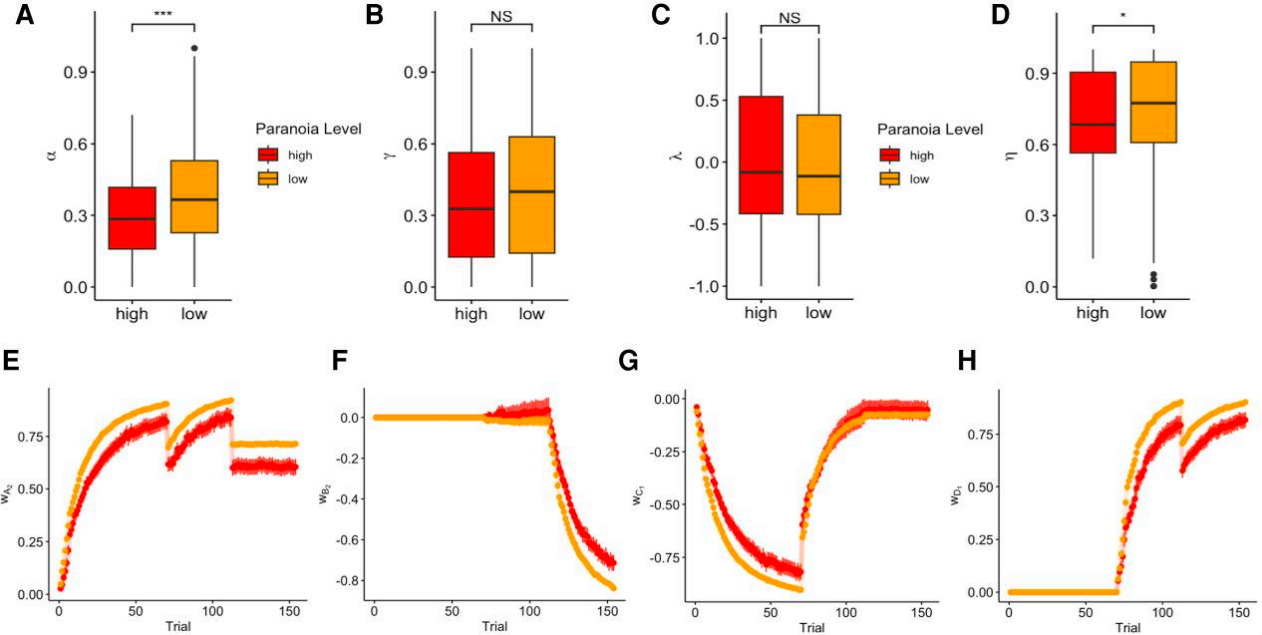
**Model parameters by paranoia level.** (**A**) Learning rate (*α*) was significantly lower in individuals with high paranoia. (**B**) The integration rule parameter (*γ*) was not significantly different between the paranoia groups. (**C**) The learning asymptote parameter for the secondary cue (*λ*) was not significantly different between paranoia groups. (**D**) Individuals with high paranoia had a lower value for the ‘forgetting’ parameter, reflecting a greater decay in cue weights in between phases. (**E**–**H**) Time series for averaged model cue weights split by high and low paranoia. Error bars show standard error of the mean. (**E**) Weights for the blocking cue are lower across the entire task for the high-paranoia group. (**F**) There is separation during Phase 3 for the weight of the blocked cue; the weight increased during A_2_B_2_+ pairing for the high-paranoia group. (**G** and **H**) Weights for the control cues; individuals with high paranoia struggled to learn all the cues, reflected in the lower cue weight for D_1_+. *P ≤ 0.05; ***P ≤ 0.001; NS: *P* > 0.05.

Given that both those with higher paranoia and those with high conviction non-paranoid delusion-like beliefs overlearned about the blocked cue (Fig. 3B and C), we examined the computational correlates of those two behaviours independently. In people with high conviction in non-paranoid delusion-like beliefs (*n* = 113), *λ*, which controls the direction of updating of the blocked cue by setting the learning asymptote for only the secondary cue, was elevated in individuals with high conviction in non-paranoid delusions [*F*(1,449) = 5.75, *P* = 0.016, $\eta_{p}^{2}$ = 0.01; Fig. 6]. This results in the secondary cue being driven towards the observed outcome attributed to the primary cue. This is the same parameter that allows for counterfactual reasoning: individuals with negative *λ* values update the secondary stimulus towards the unobserved outcome (no allergy, minus one). In contrast, participants with high paranoia (*n* = 65) had significantly lower learning rates [*α*: *F*(1,449) = 11.95, *P* < 0.001, $\eta_{p}^{2}$ = 0.03; Fig. 7]. Of note, the learning rate parameter is used in updating all stimuli throughout the task, resulting in impairments in learning across all stimuli. Individuals with high paranoia also had increased *η* values, reflecting increased ‘forgetting’ of cue weights when new phases of the task begin [*F*(1,449) = 4.69, *P* = 0.03, $\eta_{p}^{2}$ = 0.01; Fig. 7]. Plots of learning trajectories for the blocking and control cue patterns are shown in Figs 6 and 7, demonstrating the impact of these parameter differences on the learning trajectories for the blocking and control cue patterns.

One possibility is that people with paranoia simply did not comprehend the blocking task. We show that all the behavioural and computational analyses survive when we covary for the wide range achievement test score (a predictor of full-scale IQ; all analyses reported in Supplementary Table 7). Furthermore, for the PRL there was no significant difference in the number of points achieved between participants with high and low paranoia. These findings militate against the lack of comprehension explanation.

### Dissociating paranoia and other delusion-like beliefs

Finally, we sought to demonstrate selectivity of these effects to delusion-like belief contents and with regard to other symptoms. Specificity claims can be made by fractionating the sample into people high on one dimension and low on the others and vice versa.^44^ The challenge with the present data is that people who are paranoid often report more other delusion-like beliefs with high conviction, likewise hallucination-like percepts,^45^ and people with more psychosis-like symptoms often have lower mood. In addition, there are potentially multiple comparisons problems when many associations are computed. We turned to Bayesian Gaussian graphical models, a conservative approach to establishing all (un)conditional dependencies or partial correlations (*ρ*) between a set of variables.^46^ For each node within the Bayesian Gaussian graphical model, we calculate the probability of one of three hypotheses: probability for the null hypothesis, *ρ* = 0 [*P*(H0)]; probability for Hypothesis 1, *ρ* > 0 [*P*(H1)]; and probability for Hypothesis 2, *ρ* < 0 [*P*(H2)]. We fit two models, the first with paranoia, PDI and the Kamin blocking computational model parameters (*α*, *λ* and *η*); and the second model with the same variables and with the addition of depression and hallucinations.

**Figure 8:**
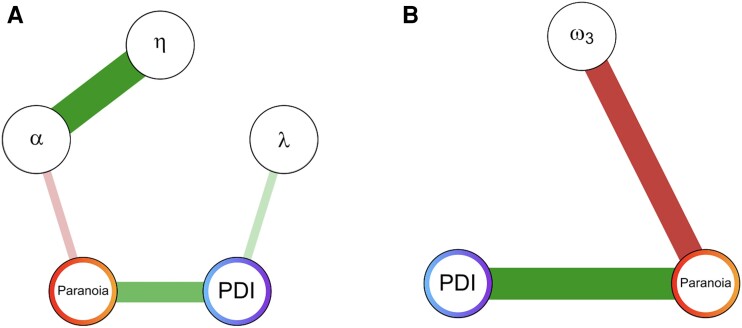
**Bayesian Gaussian graphical model^44^ visualization shows that the relationships between model parameters and delusional content [paranoia versus non-paranoid delusion-like belief conviction (PDI)] can be dissociated.** Continuous values are used for paranoia and PDI rather than groups. (**A**) Paranoia was associated with the learning rate (*α*), whereas PDI was associated with the learning asymptote of the secondary cue (*λ*). A green edge represents a positive partial correlation and a red edge a negative partial correlation. (**B**) Only paranoia was associated with the learning rate for volatility ($\omega_{3}$ averaged over blocks) in the hierarchical Gaussian filter (HGF) model.

Computational model parameters derived from blocking behaviour were differentially associated with paranoia versus non-paranoid delusion-like beliefs. Paranoia [but not non-paranoid delusion-like beliefs; *P*(H0) = 0.856] was associated with lower learning rates [*α*; *P*(H2) = 0.901], whereas non-paranoid delusion-like beliefs [*P*(H1) = 0.898], but not persecutory paranoia [*P*(H0) = 0.89], were related to non-selective learning about the blocked cue (*λ*; Fig. 8A). Hallucinations and depression did not account for the association between *λ* and PDI-non-paranoid conviction [*P*(H1) = 0.901]; however, adding these additional symptoms into the model removed the connection between *α* and paranoia [*P*(H2) = 0.800; Supplementary Fig. 2A].

Finally, we subjected the PRL modelling data to the same procedure. We found that $\omega_{3},$ the meta-volatility learning rate, was correlated specifically with paranoia [*P*(H2) = 1.0] and not PDI [*P*(H0) = 0.433] (except via the connection between paranoia and PDI). Subjecting these results to the same test of specificity, we found that they were robust to inclusion of hallucinations and depression [$\omega_{3}$-paranoia: *P*(H2) = 1.0; $\omega_{3}$-PDI: *P*(H2) = 0.714; $\omega_{3}$-depression: *P*(H0) = 0.801; $\omega_{3}$-hallucinations: *P*(H0) = 0.594; Supplementary Fig. 2B]. These results represent a dissociation of the roles of computational parameters in delusion-like beliefs with different contents: within the blocking task, *λ* was associated with non-paranoid delusion-like thinking and not paranoia, whereas within the PRL task, the meta-volatility learning rate was associated with paranoia and not with non-paranoid delusion-like beliefs (Fig. 8B).

## Discussion

Our data reveal two separate routes to delusion-like beliefs with different contents. One route involves aberrant responses to volatility and learning about change, which impairs belief formation about all cues and relates to paranoia (but not other delusion-like beliefs). The other route entails impairment to selective learning, leaving responses to control cues intact, but specifically augmenting responses to redundant cues. This learning pattern was associated with high conviction in non-paranoid delusion-like beliefs (but not paranoia).

Our data suggest that weaker blocking (learning about the causal status of the blocked cue) can arise in multiple circumstances. First, with broadly imprecise association formation, poorer learning about the blocking cue can leave room for learning about the cue that should be blocked. This occurs in people with a low learning rate; their prediction errors are incorrectly and inefficiently calibrated to what they learn. This mechanism is associated with high paranoia. Second, some people learn well about the blocking cue (A) and the other cues, including the matched control stimuli. These people learn about the blocked cue because of errant prediction error impacting the ways in which they update the elements of compound stimuli, biasing their beliefs towards the redundant stimulus. These people tended to have high conviction in their non-paranoid delusion-like beliefs. The computational model also allowed for counterfactual inference for compound trials (A causes the allergy, therefore B does not cause the allergy), providing an option for beliefs about the blocked cue to extend towards prevention of the allergy, as seen in the behaviour. However, people more convinced of their non-paranoid delusion-like beliefs were less likely to engage in this counterfactual updating. This is consistent with prior data suggesting that delusion-like ideation is related to associative learning rather than propositional inference.^21^

There was internal consistency across the task results. Participants who learned the PRL erratically also had poor learning (about the blocked and control cues) in the Kamin task [WSR versus control score: *r* = −0.19, *P* < 0.0001, 95% CI: (−0.28, −0.1)]. This was also manifest as a lower learning rate, and these learning rates were significantly correlated across tasks, and both correlated with paranoia [*α* versus $\omega_{3}$: *r* = 0.202, *P* < 0.001, 95% CI: (0.11, 0.29)].

Interestingly, in both tasks we found a better differentiation in symptom-based groups (paranoia and PDI conviction) rather than syndrome-based groups (phenotypes). This supports the idea that computerized screening might be better at detecting specific disfunctions or symptoms instead of whole diagnosis groups. Also, this aligns with some precision psychiatry scopes focusing on dimensions of individuals instead of broad diagnosis groups.^1^

Prior work with Kamin blocking tasks has related weaker blocking to distress regarding delusion-like beliefs,^6^ negative symptoms^47^ or disorganization^48^ or has found no relationship between blocking and psychosis-like symptoms.^49^ There are at least three different reasons for these differences: (i) the studies had smaller samples; (ii) their largely healthy participants might not have been experiencing sufficient conviction in their delusion-like beliefs to yield a detectable association; and (iii) the tasks used either emphasized spatial cognition and explicit reward^48^ or used linguistic stimuli,^47,49^ which might have loaded more heavily on other cognitive processes more relevant for negative symptoms and disorganization.^50^

The present data represent a key step forwards in the prediction error account of delusions. Hitherto, the account has been disparaged for failing to explain the contents of delusion by two-factor theorists who insist that prediction error processing deficits are either insufficient to explain delusions or that the prediction error deficit is the purview of reasoning problems.^51^ Our prior work on Kamin blocking has challenged the reasoning account^21^; aberrant prediction errors and not propositional reasoning during causal inferences relate to delusion-like beliefs. Here, we show that paranoia and other delusions have distinct correlates in learning mechanisms downstream of the prediction error dysfunction. This might sound like a two-factor explanation; however, we suggest that the domain of the delusion (social, regarding the self, etc.) might be in the data.^52^ For example, given a specific prediction error dysfunction that encourages erratic belief updating, paranoia may result, perhaps because we attempt to palliate that particular type of uncertainty by blaming other people.^15^ Humans find uncertainty highly aversive,^53^ and they have a need to reconcile it, even if the solution is errant.^54^ Here, we show that paranoia arises under domain general volatility beliefs, which, given that other humans are often causes and that their intentions are hard to infer, such volatility is often ascribed to them. It is also reassuring to have an enemy on which to blame experiences of uncertainty and contingency^55^ (in our prior work with patients with schizophrenia, this contention was supported by the significant associations between paranoia, task-related volatility beliefs and worry^19^). This contention also finds support in our observation that when the world became more uncertain during the COVID-19 pandemic, paranoia, conspiracy theories and erratic PRL behaviour all increased,^16^ and participants reported feeling sabotaged by the inanimate card decks in PRL.^16^ If the timing and impact of prediction error are instead focused on situations where potential causes are correlated and contiguous, such that appropriate and inappropriate conclusions are confounded, aberrant prediction error can drive attention and learning towards the inappropriate conclusion. Examination of blocking and belief updating in neuropsychological patients with monothematic delusions or paranoia consequent to brain lesions will test these hypotheses.

Given that the present results were gathered in people who are not (yet) delusional, they bear replication in people with confirmed delusional beliefs. However, there is evidence linking delusions to aberrant prediction error in our prior work and from meta-analyses. Furthermore, unusual thought content^56,57^ (delusion-like beliefs) and suspiciousness^58,59^ (paranoia) portend conversion to a first episode of psychotic illness from the clinical high risk state. In ongoing work, we are assessing whether the present behavioural and computational metrics can enhance the prediction of conversion to delusions and psychotic illness, in addition to the trajectories of those states.^60^

It is notable that these tasks and computational parameters do not distinguish phenotype groups and instead cleave to paranoia and other delusion-like beliefs. Criteria for CHR-P syndrome include several paths involving a combination of symptoms (e.g. unusual thoughts, suspiciousness, grandiosity, unusual perceptions, disorganized communication) and also include consideration of timing and symptom frequency, in addition to factors such as relationship to a relative with a psychotic disorder and deterioration of social and role functioning. Tasks and models that capture those other symptoms, combined with the present delusion-relevant metrics, will be likely to delineate the phenotype groups better. Nevertheless, the present tasks and models have utility in distinguishing delusion-like beliefs with different contents, with theoretical implications.

This combination of PRL and blocking tasks could also have translational utility. Amphetamine can engender psychosis-like states in humans.^61^ In rodents, amphetamine administration weakens blocking^12^ and induces erratic win-switching behaviour.^28,62^ These neuropsychopharmacological tools could facilitate development of targeted treatments for paranoia and delusions.^63^ Furthermore, the underlying circuitry of these effects could be uncovered; lesions to the mediodorsal thalamus in rodents induce weaker blocking,^64^ and similar lesions in primates engender erratic win-switch behaviour.^65^ The locus coeruleus projection to the medial prefrontal cortex drives erratic switching behaviour,^66^ and medial prefrontal activity weakens blocking in rodents^11^ and humans.^67^ Finally, volatility priors^68^ and aberrant prediction errors^3^ engage the dorsolateral prefrontal cortex. Triangulating the shared and unique mechanisms of these behavioural effects will deepen our understanding of the similarities and differences between paranoia and other delusions.^45^

Although the models we fitted to each task shared many features, and each was the best-fitting model for that task, in future we might fit identical models to both tasks. We thought that unwise here, because the Kamin task is deterministic (and thus would not engage the volatility and meta-volatility learning integral to the HGF and necessary for modelling the PRL task), and it involves learning about many cues concurrently. We and others have handled such complexity by adding arms to the HGF (e.g. for social and non-social stimuli), but the number necessary for the blocking task seems unwieldy. Nevertheless, a common modelling framework might facilitate the discovery of underlying mechanisms. We note that recovery of the $\omega_{3}$ parameter from PRL was significant, but could have been more compelling. Furthermore, some of the parameters at Level 2 had poor recovery [initial beliefs about rewards ($\mu_{0}^{2}$) and phasic learning rate (*κ*); these did not differ between groups, and we did not consider them further in our symptom content analyses]. Given that these parameters also influence what happens at Level 3, we should be cautious in interpreting our findings (Supplementary Table 6). The possibility remains that a better model might be identified. For example, adding a mean-reverting value to the volatility belief might identify CHR^68^ and first episode psychosis,^69^ and volatility beliefs can be recovered well from such models.^69^ In these cases, the second level of the model is sometimes fixed.^68^ Fixing parameters (or changing parameter ranges, as observed here) can alter the specific parameters related to an illness or symptom, because behavioural variance will be assumed by the parameters that remain and whose values can vary, hence the focus on $\omega_{3}$ here (versus $\mu_{0}^{3}$ previously^16^). Furthermore, the tasks upon which these psychosis-sensitive model effects are based differ markedly from the present task.^68^ This PRL task is extremely volatile from the start, has more response options to track, and injects unexpected uncertainty midway through. Finally, models with different structure sometimes fit different groups of participants better^69^ (although in the study by Hauke *et al.*^69^ CHR and controls did not differ, as we observed). That is not the approach taken here, where we emphasize the continuum of delusion-like belief. Nevertheless, future work should take these approaches and ought to reconcile the present findings with other work on HGF, psychosis and delusion-like belief, despite task and sample differences.

Recent meta-analysis of prediction error functional imaging studies localized domain general prediction errors to the midbrain, striatum, insula and prefrontal cortex.^2^ Precision weighting and social prediction error shared territory in the insula.^2^ We predict that non-paranoid delusions would involve aberrant frontostriatal prediction errors, and paranoia would involve errant precision weighting of prediction error in the insula. Within meso-accumbal dopamine pathways, states and rates of change of state are coded in medial and lateral ventral tegmental area dopamine neurons, respectively.^70^ This division might prove relevant for non-paranoid and paranoid delusion-like beliefs.

Crucially, cognitive behavioural therapy for psychosis may not alleviate all delusions,^71^ but specific worry interventions do mollify paranoia,^20,72^ and worry mediates the relationship between paranoia and learning from volatility.^19^ The quantitative behavioural metrics we have identified might serve as predictors or correlates of therapeutic responses. A successful therapy for paranoia, but perhaps not for other delusions, reinforces our claim that the psychological processes underlying paranoid and other delusion-like beliefs might be dissociable. However, our data suggest that aberrant prediction errors underlie both, and the differential impact of those errors on subsequent processing, learning rates and cue combination provide the distinct contents of delusion-like beliefs.

## Supplementary Material

awae122_Supplementary_Data

## Data Availability

The data and model likelihood functions that support this paper are available at https://github.com/rosarossig/capr_project/.
