# Supplementary material for: Different learning aberrations relate to delusion-like beliefs with different contents
Source: Brain. 2024 Apr 18;147(8):2854–66. doi: 10.1093/brain/awae122 (PMC11292907; doi:10.1093/brain/awae122)
Supplement: awae122_Supplementary_Data [file awae122_supplementary_data.pdf]

Supplementary Information

Screening

Each potential participant attended an initial screening session where they gave informed consent, demographic data were acquired, and the screener from the structured interview for psychosis risk syndromes (SIPS) was conducted they were then be classified either as Clinical High Risk (CHR, those meeting criteria for a progressive psychosis-risk syndrome) or control. The SIPS is the most commonly used interview in the US for assessing psychosis-risk syndromes and has established predictive validity for conversion to psychosis, specificity, and inter-rater reliability. Participants will be deemed at CHR for psychosis if they meet criteria for one or more (of 3) of the primary SIPS psychosis-risk syndromes at a progressive (recently emergent or escalating) or persistent designation.

Baseline

All participants then attended a baseline session consisting of: (1) a clinical assessment battery (remainder of SIPS, Structured Clinical Interview for DSMV disorders, SCID) including a socio-occupational functioning interview and self-report measures; and (2) the computerized assessment of psychosis risk (CAPR) battery (other tasks reported elsewhere, and in future publications), as well as (3) tasks necessary to complete the North American Prodrome Longitudinal Study (NAPLS) risk calculator. Following the baseline, control participants were classified as a help-seeking control (HSC) or healthy control (HC), based on SCID diagnoses. In addition, interviewers gathered information on traumatic brain injuries, developmental history, medical concerns, and psychiatric history. Weekly clinical consensus meetings were conducted to confirm SIPS ratings and diagnoses, to ensure that the instrument was used uniformly across all sites.

Behavioral tasks

Kamin Blocking Task

Initially, some participants received a version of the task where the control stimuli were mis-paired. Specifically, participants who received in the incorrect version had F- paired with I+ (IF+), while participants who received the correct version had F- paired with E- (EF-), and I+ alone (Supplementary Table 1). Since this coding error did not impact the blocking (AB) or control (CD) trials, we chose to process and analyze all data, including with model fitting.

Probabilistic Reversal Learning Task

Reward contingencies change from 90%, 50%, and 10% chance of reward to 80%, 40%, and 20% between the first and second halves of the task ("contingency transition"; block 1 = 80 trials, 90–50-10%; block 2 = 80 trials, 80–40-20%).

Questionnaires

Peter's Delusions Inventory – 21 (PDI-21)

The PDI-21 is a 21 item, dichotomous (Yes/No) self-report questionnaire to assess delusional symptoms in the general population. The higher the score, the greater the delusional symptoms. For each item, three follow up questions of 5 categories of response (1 to 5) are provided corresponding to the subscales of the degree of conviction, preoccupation, and distress associated with each endorsed belief. The PDI-21 has several themes that tap into paranoia, such as suspiciousness (items 1 and 3), persecution (items 4 and 5), and paranoid thoughts (items 13 and 15). We separated the items that were included in those 3 themes to create a separate PDI-paranoia score, and used the remaining questions to create a PDI-non-paranoid delusion score (16 items in total). Each one of the subscores then has mean distress, mean preoccupation, and mean conviction per endorsed belief. The PDI does not have a clinical cutoff, so the "high" PDI group had PDI-non paranoid delusion conviction scores above the 75% quantile for the sample.

Revised Green et al Paranoid Thoughts Scale

A self-report scale used to capture paranoia—the belief that others have bad intentions towards us—along the continuum from health to illness, and across diagnoses. A score of 11 or above indicates a clinical level of persecutory paranoia and is used as the cutoff for the high paranoia group. While our primary analysis uses only this clinical cutoff, we also plot behavioral and parameter analyses from the PRL using the other GPTS grouping levels (Supplementary Figure 3). Scores below 5 indicate low paranoia, 5-10 is elevated paranoia, 11-17 is moderately severe paranoia, 18-27 is severe paranoia, and 28 and above is very severe paranoia[1].

Hierarchical Gaussian Filter (HGF)

The Hierarchical Gaussian Filter (HGF) toolbox v5.3.1 is freely available for download in the TAPAS package at https://translationalneuromodeling.github.io/tapas [2-4]. We installed and ran the package in MATLAB and Statistics Toolbox Release 2022b (MathWorks ®, Natick, MA).

We estimated perceptual parameters individually for the first and second halves of the task (i.e., for trials 1-80 and then trials 81-160). Each participant's choices (i.e., deck 1, 2, or 3) and outcomes (win or loss) were entered as separate column vectors with rows corresponding to trials. Wins were encoded as '1', losses as '0', and choices as '1', '2', or '3'. We selected the autoregressive 3-level HGF multi-arm bandit configuration for our perceptual model and paired it with the softmax-mu03 decision model.

Kamin Blocking Model

For single cue trials, the model computes a prediction for the trial using the value of the weight matrix for that cue ($w_{t,i}$, where i is the cue type) given by:

$$\hat{r} = w_{t,i} \qquad \textit{Eq. 1}$$

The prediction is then used to generate a prediction error for this trial:

$$\delta = r_t - \hat{r} \qquad \textit{Eq. 2}$$

where r_t is the outcome (1 = allergy, -1 = no allergy) for this trial. This results in a smaller prediction error when the prediction is close to the observed outcome. The weight for the observed cue is then updated according to a classic Q-learning rule:

$$w_{t+1,i} = w_{t,i} + \alpha\delta \qquad \textit{Eq. 3}$$

Where α is the constant learning rate parameter that governs how much the prediction error contributes to the update. A lower value corresponds to a smaller update – which corresponds to decreased learning. Weights for the non-observed cues are not updated, so:

$$w_{t+1,j} = w_{t,j} \qquad \textit{Eq. 4}$$

Trials with compound stimuli follow a distinct update rule. First, the stimulus is separated into the cues, which are categorized as primary or secondary depending on the value of the associated weight. These weights are given by:

$$w_p = max(w_i, w_j) \qquad \textit{Eq. 5}$$

$$w_s = min(w_i, w_j) \qquad \textit{Eq. 6}$$

The primary cue is the cue that is more likely to cause allergy (as determined by the cue weight) while the secondary cue is the cue that is less likely to cause allergy. For a compound stimulus we do combine the cues to generate an overall prediction:

$$\hat{r} = w_{t,p} + \gamma w_{t,s} \qquad \textit{Eq. 7}$$

This integration rule allows for a range of cue integration strategies by controlling the parameter γ. Individuals with $\gamma = 0$ use only the primary cue weight to determine the prediction, corresponding to a max-estimation. Individuals with $\gamma = 1$ add the cues, which corresponds to an additive integration rule (this is used in RW and other blocking models). We fit γ as a free parameter in order to capture a range of behavior.

The prediction error for the primary cue is simply the classical update rule using the prediction from Equation 7:

Eq. 8

$$\delta_p = r_t - \hat{r}$$

A max-estimation model uses only the value the primary cue in the update, so the value of the secondary cue does not impact the update of the primary cue. For a pure additive model, the primary stimulus is updated using the summation of the other cues, reflecting how the primary cue value may be influenced by the value of the secondary cue. The update for the primary cue is then:

$$w_{t+1,p} = w_{t,p} + \alpha\delta_p$$

Eq. 9

The update to the secondary cue is where the possibility of counterfactual updating can be explored. We define the update to the secondary cue to be:

$$\delta_s = \lambda_t r_t - w_{t,s}$$

Eq. 10

First, the prediction used to compute the PE uses only the value of the secondary cue – the secondary cue is the irrelevant cue, and as such the integrated prediction is not used. This concept allows us to incorporate counterfactual updating of the secondary cue into the model using λ_t, given by:

$$\lambda_t = \begin{cases} \lambda & w_p \geq 0 \\ 1 & w_p < 0 \end{cases}$$

Eq. 11

The piecewise nature of λ_t allows us to incorporate the task-specific logic: for individuals who do counterfactually update the secondary stimulus, they only do this for compounds where they expect an allergy (the primary cue is likely to cause allergy). It makes no sense to counterfactually update the secondary stimulus in a compound that doesn't cause allergy, since then the compound should cause allergy.

The update equation for the secondary stimulus is then given by:

$$w_{t+1,s} = w_{t,s} + \alpha\delta_s$$

Eq. 12

An additional feature noted in the data (Figure 3D-G) was that individuals appeared to "forget" the values of the cues when the task shifted to a new phase. Of note, there was no explicit separation between the phases, but the cues did change, signifying a new phase. To incorporate this feature into

Eq. 13

the model, we introduce a “forgetting” parameter that decays the weights on the first trial of a new phase:

$$w_{t,all} = w_{t,all}\eta$$

η can range between 0 and 1, and all cue weights are discounted the same amount towards 0. A value of 1 corresponds to someone who does not forget the values at the new phase.

Parameter recovery on simulated data

To validate our models and ensure that the model sufficiently captures the behavioral features of interest[5,6], we simulated choice data for both the PRL and the Kamin blocking task using each individuals’ fitted parameter sets. We simulated choice data 15 times for the PRL task and 50 times for the blocking task, and refit the model on this simulated data to created simulated parameter sets. We can then look at how well the parameters can be recovered by examining the correlations between fitted and simulated parameters. This is crucial for model validation; as maximum-likelihood estimation of parameters can give slightly different estimates every time the model is fit. Correlations between each model parameter and its simulated version are shown in Supplementary Table 6, and plots of these correlations are shown in Supplementary Figure 1 and 4. All parameter effects of interest could be observed using the simulated parameters instead of the actual parameter fitted values (Supplementary Table 8).

Statistics

We implement Bayesian Gaussian Graphical Models (BGGM [7]) – this model calculates all the statistical dependencies in a set of variables to estimate the relation between any two nodes, adjusting by all the rest of nodes. All connections in the network are estimated with a credible interval. Thus, we consider credible all the connections where the 0 is outside the 95% credible interval. In addition to the credible intervals, we estimated the probability for the null hypothesis, i.e., a pair of nodes are not connected.

We did so with the function *select()* and the exhaustive option from the same R BGGM package. This option calculates the probability of three hypotheses: H0: partial correlation $\rho = 0$; H1: $\rho > 0$; and H2: $\rho < 0$. We visualize only these connections with the package *qgraph*[8].

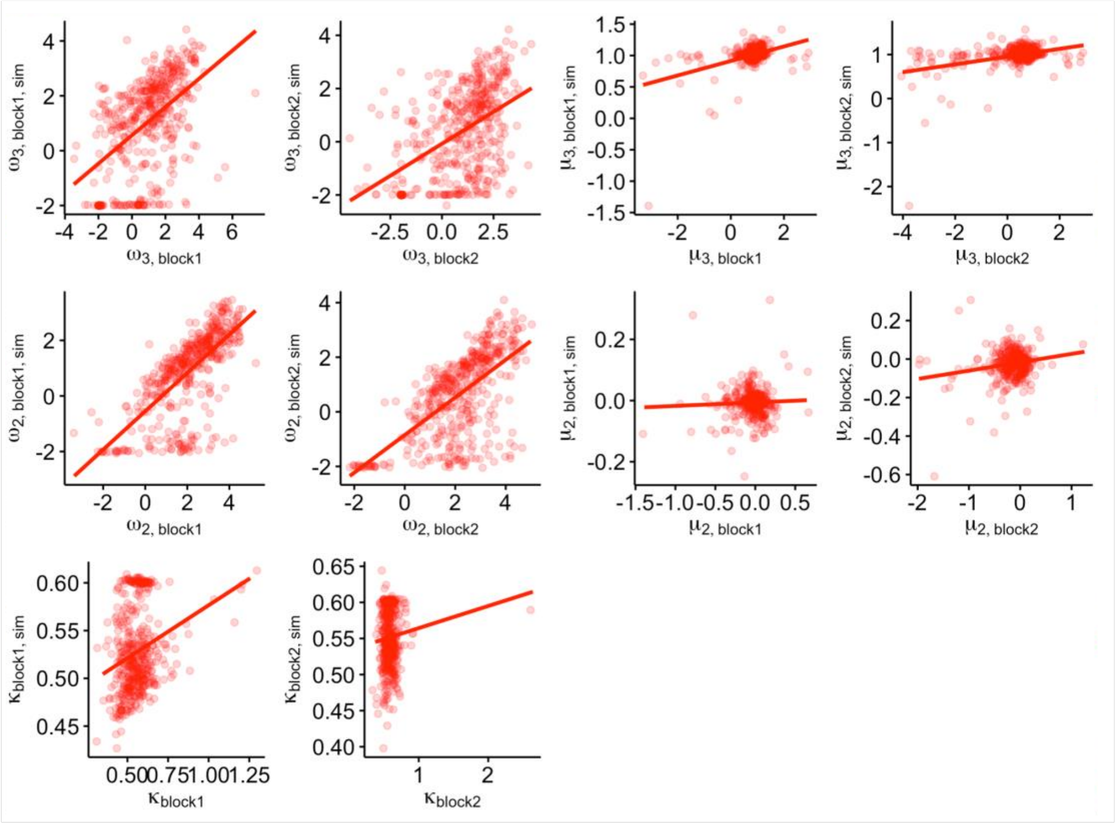

Supplementary Figure 1 – correlations between fitted and simulated parameters: PRL

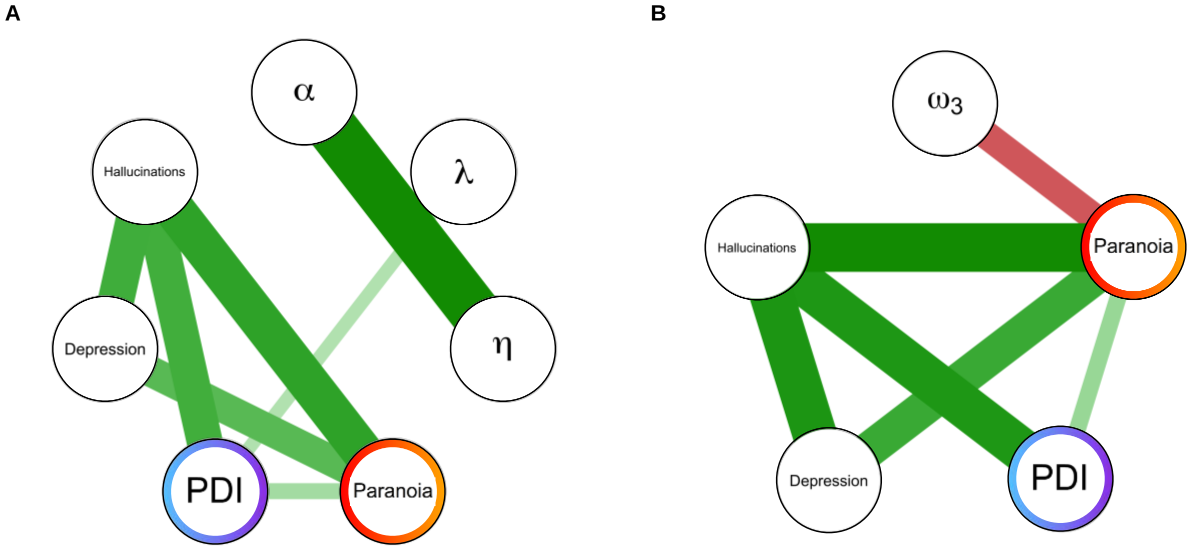

Supplementary Figure 2

A) Hallucinations (LSHS) and depression (CESD) scores did not account for the associations between λ and non-paranoid delusional conviction beliefs (PDI). Adding hallucinations and depressions did eliminate the relationship between α and paranoia, suggesting that depression and hallucinations may contribute to the observed relationship between learning rates and paranoia. **B)** The relationship between volatility learning rates and paranoia remains even when accounting for depression and hallucinations.

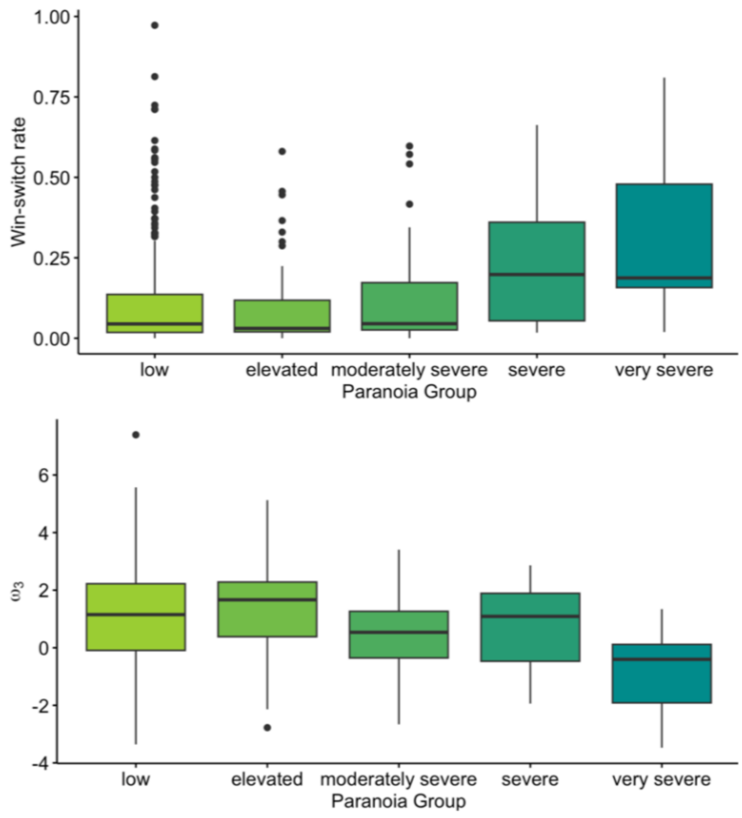

Supplementary Figure 3

WSR and ω_3 values show a consistent pattern across paranoia groups used in the R-GPTS. We note that the clinical cutoff used in the text to delineate high from low paranoia includes individuals with moderately severe, severe, and very severe GPTS scores.

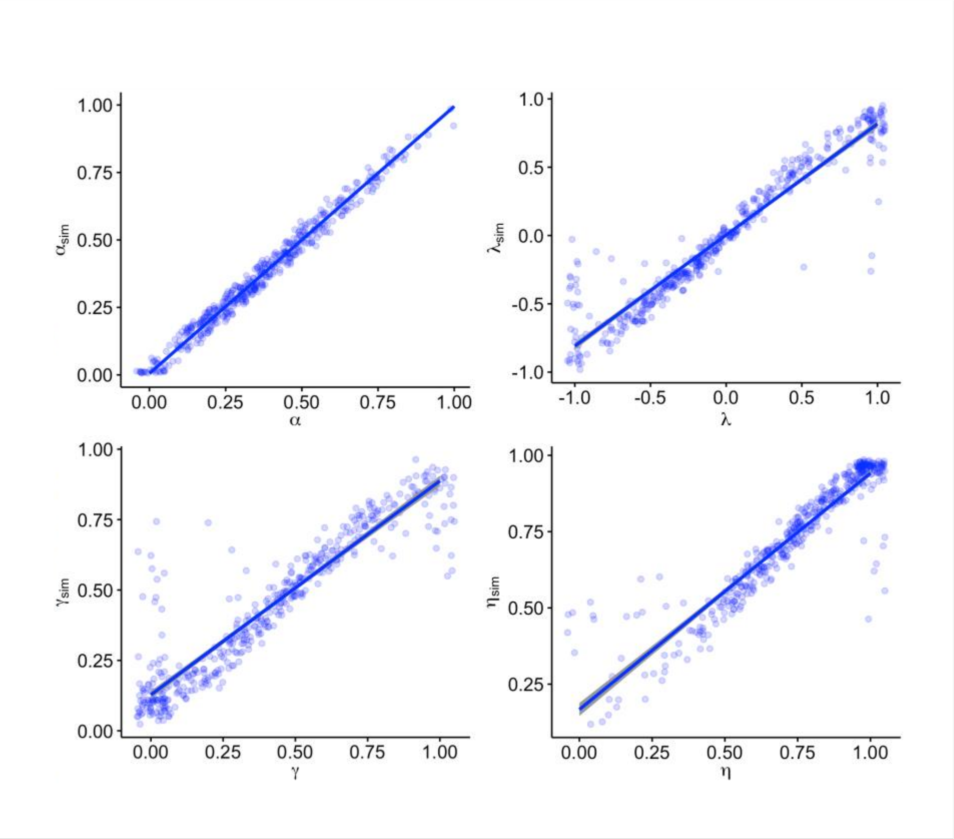

Supplementary Figure 4 – correlations between fitted and simulated parameters: blocking

Supplementary Table 1 – Food stimuli table

Stage 1	Stage 2	Stage 3	Role
A_1+	A_1B_1+	B_1+	Violation of blocking
A_2+	A_2B_2+	B_2-	Confirmation of blocking
C_1-	C_1D_1+	D_1+	Confirmation of blocking control
C_2-	C_2D_2+	D_2-	Violation of blocking control
F-	EF-	EF-	No allergy control*
I+	I+	I+	Consistent Allergy*
J-	J-	J-	Consistent No Allergy

Letters represent food stimuli, + indicates presence of allergy, - indicates no allergy. The roles of that particular food stimuli played were randomized and counterbalanced across participants.

*Due to a coding error, a total of 305 participants experienced different control cues at Phase 3, They saw E- and IF+ trials. These participants did not differ in their strength of blocking. Furthermore, the modeling analysis takes into account all trials a participant experiences.

Supplementary Table 2 – Model comparison

Model	Parameters	Integration Rule	XP	PXP
RW1	$\alpha, \gamma, \lambda, \eta, u$	Flexible	0	<0.000001
**RW2**	$\boldsymbol{\alpha, \gamma, \lambda, \eta}$	**Flexible**	**1**	**1**
RW3	α, γ, λ	Flexible	0	<0.000001
RW4	α, λ	Additive	0	<0.000001
RW5	α, λ	Max	0	<0.000001

Supplementary Table 3 – Sample characteristics

**Demographics**	CHR (N = 181)	HSC (N = 161)	HC (N = 110)
Age	23.34 (4.29)	23.67 (4.03)	23.56 (4.06)
% Female	66.40	66.46	54.55
% High Paranoia	26.52	8.07	3.64
% High PDI-C	29.28	22.98	20.91
% White	54.44	53.75	50.00
% Black/African American	12.22	13.75	14.55
% Asian	18.33	22.50	28.18
% Native American	1.11	1.25	0.00
% Multiracial	11.67	6.88	7.27
% Race Unknown	2.22	1.88	0.00
% Hispanic/Latino (any race)	15.64	11.88	6.36

Supplementary Table 4 – Additional analysis

Measure	Symptom	Behavioral Measure	Statistics
LSHS	Hallucinations	WSR	F(1, 440) = 1.44, p = 0.23
MAPSR	Depression	WSR	F(1, 440) = 0.19, p = 0.66
CESD	Depression	WSR	F(1, 440) = 3.66, p = 0.06
LSHS	Hallucinations	Blocking Score	F(1, 440) = 0.23, p = 0.63
MAPSR	Depression	Blocking Score	F(1, 440) = 0.18, p = 0.67
CESD	Depression	Blocking Score	F(1, 440) =1.32 , p = 0.25
LSHS	Hallucinations	Control Score	F(1, 440) = 1.86, p = 0.17
MAPSR	Depression	Control Score	F(1, 440) = 0.09, p = 0.77
CESD	Depression	Control Score	F(1, 440) = 1.18, p = 0.28

Supplementary Table 5 – HGF priors

Parameter	Mean	Variance	Estimation Space
μ_2^0	0	1	Native
μ_3^0	1	1	Native
κ	.6	.1	Log
ω_2	-2	4	Native
ω_3	-2	4	Native
ϕ_2	0.4	1	Logit
ϕ_3	0.2	1	Logit

Initial parameters are consistent with prior work[15,16] with the exception of the variance on ω_2 and ω_3. We widened the prior on those parameters to avoid the estimations being stuck on the bounds.

Supplementary Table 6 – Parameter recovery

HGF Parameter	Correlation between fitted parameter and simulated parameter	Cohen's f^2
$\omega_{3,\text{block 1}}$	r = 0.504, p < 0.0001	**f^2 = 0.34**
$\omega_{3,\text{block 2}}$	r = 0.477, p < 0.0001	**f^2 = 0.30**
$\mu^0_{3,block\ 1}$	r = 0.437, p < 0.0001	**f^2 = 0.24**
$\mu^0_{3,block\ 2}$	r = 0.391, p < 0.0001	**f^2 = 0.18**
$\omega_{2,block\ 1}$	r = 0.695, p < 0.0001	**f^2 = 0.93**
$\omega_{2,block\ 2}$	r = 0.634, p < 0.0001	**f^2 = 0.67**
$\mu^0_{2,block\ 1}$	r = 0.045, p = 0.34	**f^2 < 0.01**
$\mu^0_{2,block\ 2}$	r = 0.194, p < 0.0001	**f^2 = 0.04**
$\kappa_{block\ 1}$	r = 0.254, p < 0.0001	**f^2 =0.07**
$\kappa_{block\ 2}$	r = 0.090, p = 0.058	**f^2 < 0.01**

**RL Parameter**	**Correlation between fitted parameter and simulated parameter**	**Cohen's f^2**
α	r = 0.99, p < 0.0001	**f^2 = 759.54**
γ	r = 0.93, p < 0.0001	**f^2 = 6.50**
λ	r = 0.95, p < 0.0001	**f^2 = 10.18**
η	r = 0.93, p < 0.0001	**f^2 = 6.82**

Supplementary Table 7 – ANCOVAs using WRAT raw score as covariate

Dependent Variable	Independent Variable	Statistics
WSR	Paranoia level	F(1,448) = 12.64, p < 0.001
Blocking score	Paranoia level	F(1,448) = 5.64, p = 0.018
Control score	Paranoia level	F(1,448) = 5.54, p = 0.019
Omega-3 block 1	Paranoia level	F(1,446) = 9.93, p = 0.002
Omega-3 block 2	Paranoia level	F(1,446) = 6.72, p = 0.01
Alpha	Paranoia level	F(1,448) = 10.18, p =0.002
Eta	Paranoia level	F(1,448) = 3.19, p = 0.07
Blocking score	PDI-C level	F(1,448) = 7.73, p = 0.006
Lambda	PDI-C level	F(1,448) = 5.93, p = 0.015

Supplementary Table 8 – behavioral effects using simulated parameters

**Effect**	**Parameter**	**Model**	**Statistics**
ω_3-paranoia effect	$\omega_{3,sim}$	PRL/HGF	F(1,448) = 12.11, p = 0.00055
α-paranoia effect	α_{sim}	Blocking	F(1,449) = 11.72, p = 0.0007
η-paranoia effect	η_{sim}	Blocking	F(1,449) = 4.32, p = 0.038
λ-PDI-C effect	λ_{sim}	Blocking	F(1,449) = 6.48, p = 0.011

Supplementary Table 9 – PDI-paranoid subscale analysis

Groups	WSR effect	Om.3 effect
High paranoia: above clinical cutoff on GPTS AND above 75% quantile on PDI-paranoia conviction score	F(1,450) = 20.6, p < 0.0001	F(1,448) = 23.4, p < 0.0001

References

1. Freeman D, Loe BS, Kingdon D*, et al*. The revised Green et al., Paranoid Thoughts Scale (R-GPTS): psychometric properties, severity ranges, and clinical cut-offs. *Psychol Med*. Jan 2021;51(2):244-253. doi:10.1017/S0033291719003155
2. Frassle S, Aponte EA, Bollmann S*, et al*. TAPAS: An Open-Source Software Package for Translational Neuromodeling and Computational Psychiatry. *Front Psychiatry*. 2021;12:680811. doi:10.3389/fpsyt.2021.680811
3. Mathys C, Daunizeau J, Friston KJ, Stephan KE. A bayesian foundation for individual learning under uncertainty. *Frontiers in human neuroscience*. 2011;5:39. doi:10.3389/fnhum.2011.00039
4. Mathys CD, Lomakina EI, Daunizeau J*, et al*. Uncertainty in perception and the Hierarchical Gaussian Filter. *Frontiers in human neuroscience*. 2014;8:825. doi:10.3389/fnhum.2014.00825
5. Wilson RC, Collins AG. Ten simple rules for the computational modeling of behavioral data. *Elife*. Nov 26 2019;8doi:10.7554/eLife.49547
6. Palminteri S, Wyart V, Koechlin E. The Importance of Falsification in Computational Cognitive Modeling. *Trends Cogn Sci*. Jun 2017;21(6):425-433. doi:10.1016/j.tics.2017.03.011
7. Williams DR, Rast P, Pericchi LR, Mulder J. Comparing Gaussian graphical models with the posterior predictive distribution and Bayesian model selection. *Psychol Methods*. Oct 2020;25(5):653-672. doi:10.1037/met0000254
8. Epskamp S, Cramer, A. O., Waldorp, L. J., Schmittmann, V. D., Borsboom, D. qgraph: Network Visualizations of Relationships in Psychometric Data. . *Journal of Statistical Software*. 2012;48(4):1-18. doi:https://doi.org/10.18637/jss.v048.i04
